# Functional Dissection of the TBK1 Molecular Network

**DOI:** 10.1371/journal.pone.0023971

**Published:** 2011-09-08

**Authors:** Adriana Goncalves, Tilmann Bürckstümmer, Evelyn Dixit, Ruth Scheicher, Maria W. Górna, Evren Karayel, Cristina Sugar, Alexey Stukalov, Tiina Berg, Robert Kralovics, Melanie Planyavsky, Keiryn L. Bennett, Jacques Colinge, Giulio Superti-Furga

**Affiliations:** CeMM - Research Center for Molecular Medicine, Austrian Academy of Sciences, Vienna, Austria; Kantonal Hospital St. Gallen, Switzerland

## Abstract

TANK-binding kinase 1 (TBK1) and inducible IκB-kinase (IKK-i) are central regulators of type-I interferon induction. They are associated with three adaptor proteins called TANK, Sintbad (or TBKBP1) and NAP1 (or TBKBP2, AZI2) whose functional relationship to TBK1 and IKK-i is poorly understood. We performed a systematic affinity purification–mass spectrometry approach to derive a comprehensive TBK1/IKK-i molecular network. The most salient feature of the network is the mutual exclusive interaction of the adaptors with the kinases, suggesting distinct alternative complexes. Immunofluorescence data indicated that the individual adaptors reside in different subcellular locations. TANK, Sintbad and NAP1 competed for binding of TBK1. The binding site for all three adaptors was mapped to the C-terminal coiled-coil 2 region of TBK1. Point mutants that affect binding of individual adaptors were used to reconstitute TBK1/IKK-i-deficient cells and dissect the functional relevance of the individual kinase-adaptor edges within the network. Using a microarray-derived gene expression signature of TBK1 in response virus infection or poly(I∶C) stimulation, we found that TBK1 activation was strictly dependent on the integrity of the TBK1/TANK interaction.

## Introduction

Antiviral immune responses depend on the rapid production of proinflammatory cytokines and type-I interferons (IFNs). Type-I interferons are mainly regulated at the transcriptional level. After viral infection, pattern recognition receptors sense the presence of viral nucleic acids and trigger a series of signal transduction events that lead to the transcriptional activation of the IFN-β promoter [1]. Transcription of the IFN-β gene requires the intricate interplay of several transcription factors, including IRF3/IRF7, ATF-2/c-Jun and members of the NF-κB family [2].

TANK-binding kinase 1 (TBK1) and I-κB kinase ε (IKK-ε, also called IKK-i) are pivotal regulators of type-I interferon production: With the exception of plasmacytoid dendritic cells, most cells that are deficient for both TBK1 and IKK-i fail to produce type-I interferons in response to viral infection [3], [4]. Analyses of cells that are single knockouts for TBK1 and IKK-i suggest a certain level of redundancy between TBK1 and IKK-i [3], although loss of TBK1 alone seems to have a more profound impact on type-I interferon induction than loss of IKK-i alone [3]. This is highlighted by the more recent observation that the interferon response to double-stranded DNA depends exclusively on TBK1 and not on IKK-i [5].

TBK1 and IKK-i are referred to as the “non-canonical I-κB kinases” as they are most closely related to the so called canonical I-κB kinases (IKKs) IKK-α and IKK-β that regulate the activity of transcription factors of the NF-κB family. TBK1 and IKK-i have been shown to activate transcription factors of the IRF family, mainly IRF3, by phosphorylation [6]. Phosphorylation by TBK1 occurs in the C-terminal domain of IRF3, mainly at serines 386 and 396, and triggers the dimerization and nuclear translocation of IRF3 [7]. Alternative substrates of TBK1 include the DEAD-box helicase DDX3X [8], [9] and phosphorylation of DDX3X is thought to promote IFN-β transcription, but the underlying mechanism is still poorly understood.

Affinity purification of TBK1 protein complexes led to the copurification of three adaptor proteins of TBK1 named TANK, Sintbad and NAP1 [10]. TANK was originally identified as a TRAF-binding protein with both stimulatory and inhibitory roles [11]. The connection between TANK and TBK1 (TANK-binding kinase 1) only became apparent when TBK1 was found associated with TANK in a yeast-two-hybrid screen for TANK-binding proteins [12]. Likewise, NAP1 was first isolated in a yeast-two-hybrid screen for NAK1- (or TBK1-) associated proteins [13]. Sintbad, on the contrary, was first found by large-scale proteomics effort (and named TBKBP1) [10] and later on characterized in more detail based on its sequence homology to NAP1 [14]. TANK, NAP1 and Sintbad share a common region which mediates association with TBK1 [14]. Loss-of-function experiments using RNAi indicate that all three adaptors are required for production of type-I interferons in response to viral infection [14], [15], [16]. However, this view was challenged by the recent observation that TANK-deficient mice have no apparent defect in type-I interferon production [17].

Despite the fact that these adaptors are known to be relevant for the TBK1 pathway, their respective capability to influence TBK1 function through liaising TBK1 with other components of the cellular machinery warrants a dedicated effort. We embarked on a systematic screen for proteins that are associated with TBK1, IKK-i, TANK, Sintbad or NAP1. We find that TANK, Sintbad and NAP1 form alternative complexes with TBK1 that are localized to distinct subcellular compartments. TANK, Sintbad and NAP1 all bind to the coiled-coil 2 in TBK1 and compete for TBK1 binding, suggesting that they may act as recruiting adaptors. Deletion or mutation of the coiled-coil 2 has no effect on TBK1 activity upon overexpression, but leads to a profound impairment when re-introduced into TBK1-deficient cells, indicating that the TBK1 adaptors are required for TBK1 activation under physiological conditions.

## Results

**Figure 1:**
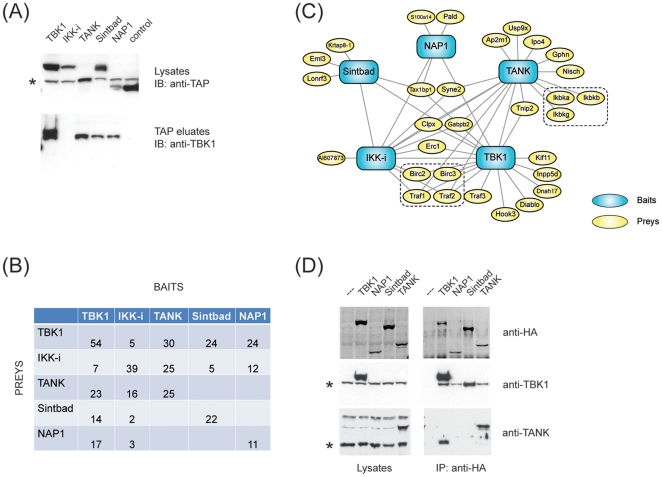
Protein networks associated with TBK1, IKK-i, TANK, Sintbad and NAP1. (A) RAW264.7 cells stably expressing GS-TAP-tagged TBK1, IKK-I, TANK, Sintbad or NAP1 were purified by tandem affinity purification. Lysates and eluates were analyzed by immunoblotting as indicated. (*) indicates a non-specific band detected by the TAP-specific antiserum. (B) Representation of the number of unique peptides identified in each mass spectrometry run (average of 2 technical repeats). Bait proteins are listed in columns, while prey proteins identified by MS are listed in rows. (C) Schematic representation of the protein-protein interaction network assembled around TBK1, IKK-i and the adaptor proteins TANK, Sintbad and NAP1. Baits are represented in blue, preys are represented in yellow. (D) NIH3T3 cells stably expressing Strep-HA-tagged TBK1, NAP1, Sintbad or TANK were lysed and subjected to immunoprecipitation using anti-HA agarose. Lysates and eluates were analyzed by immunoblotting for anti-HA.11 (Covance), anti-TBK1 (Cell Signaling) or anti-TANK (custom made). (*) indicates the band representing endogenous TBK1 and TANK, respectively.

In order to systematically identify interacting proteins of TBK1, IKK-i and their adaptors TANK, Sintbad and NAP1, we infected the murine macrophage cell line RAW264.7 with retroviral constructs leading to stable and homogenous expression of the tagged proteins at levels comparable to the endogenous situation. Next, using these cells, we performed tandem affinity purification [18]. Protein complexes were purified and complex composition was analyzed by an unbiased protein mass spectrometry approach. TBK1 and IKK-i were reciprocally copurified with TANK, Sintbad and NAP1 (Fig. 1A, B) as previously shown [8], [10], suggesting that robust interactors of TBK1 and IKK-i were captured in a faithful and reliable fashion.

In addition, we found both known and intriguing novel interactors for each of the baits used (Table S1). The specificity of interacting proteins for each bait protein was assessed by statistical evaluation against a large laboratory database of 150 affinity purifications using unrelated proteins as baits. The resultant dataset contains the baits and 30 high confidence interactors (Table S1). To obtain a global topological overview of the TBK1/IKKi network, all proteins from the filtered dataset were integrated into a protein-protein interaction network (Fig. 1C). As the analysis was centered around proteins known to interact with each other, the resulting network is coherent and shows both commonly shared and distinct interactors. The network was enriched for proteins with enzymatic activity, most notably kinases (IKKA, IKKB), E3 ligases and ubiquitin-specific proteases (BIRC2, BIRC3, TRAF3, LONRF3, USP9X). This confirms that the bait proteins are part of signal transduction networks that are embedded in a dense regulatory circuit of posttranslational modifications.

A good proportion of the interactors were previously found associated with the bait proteins or functionally implicated in innate immune signaling, validating the overall experimental strategy. Moreover, we identified several novel interactors with no previous connection to TBK1/IKKi signaling. While TANK, present here both as a bait and as a prey of TBK1 and IKKi, was known to associate with the regulatory subunit of the canonical IKK complex (IKK-γ or NEMO) [19], [20], it has not been reported to interact with the entire IKK complex comprising IKK-α, IKK-β and NEMO (Fig. 1C). TRAF3 was identified as an interactor of TBK1 and is believed to be a critical upstream activator of type-I interferon production [21]. In addition to TRAF3, we find two other TRAF family members (TRAF1 and TRAF2) associated with TBK1 and IKK-i [22]. cIAP1 and cIAP2 (encoded by the genes BIRC3 and BIRC2), which were identified as interactors of both TBK1 and IKK-I (Fig. 1C), are E3 ligases which were originally found associated with TRAF1 and TRAF2 [23] and function in the TNFR1 pathway. More recently, cIAP1/2 have also been implicated in the IRF pathway where they mediate antiviral responses [24]. Tax1bp1 which we find associated with TBK1, IKK-i, Sintbad and NAP1 (but not TANK), is a ubiquitin-binding protein that dampens TNFR1 signaling via the E3 ligase Itch [25]. Recent evidence also points towards a role of Tax1bp1 in antiviral responses [26]. Finally, we identified AI607873 which belongs to the HIN200 family of proteins as interactor of IKK-i. The founding member of this family is AIM2 [27], [28], [29], [30] which acts as a DNA sensor for the inflammasome and, as AI607873, is comprised of an N-terminal pyrin domain and a C-terminal DNA binding domain (HIN domain). Whether or not AI607873 has a role in innate immune sensing of foreign DNA is not known. Validation of the different novel components of the TBK1/IKKi network will be the subject of future studies. In this initial report, we focus on the functional dissection and rationalization of the interaction logic of the bait components TBK1, IKKi, TANK, Sintbad and NAP1.

Previous studies consistently suggested that TBK1 and IKKi form a multiprotein complex in which their adaptors TANK, Sintbad and NAP1 are obligate components [10], [14], [31]. Our redundant network analysis in which all components were used as baits and found as preys unequivocally suggested a different organization: While TBK1 and IKK-i were each found to be associated with the adaptors TANK, Sintbad and NAP1, none of the adaptors were copurified with one other (Fig. 1B). In line with this observation, TANK, Sintbad and NAP1 were found associated with distinct sets of interaction partners (Fig. 1C). This indicates that TBK1 or IKK-i may not form a single core complex containing TANK, Sintbad and NAP1, but instead may form alternative complexes with each of the adaptors.

To test this hypothesis, we created stable cell lines expressing HA-tagged versions of TBK1, NAP1, Sintbad and TANK. Immunoprecipitation of each of those bait proteins using anti-HA agarose lead to coprecipitation of TBK1 (Fig. 1D), confirming the TAP results. Of note, TANK was only coprecipitated with TBK1, but neither with NAP1 nor with Sintbad (Fig. 1D). This supports the notion that the TBK1 adaptors may bind in a mutually exclusive fashion.

**Figure 2:**
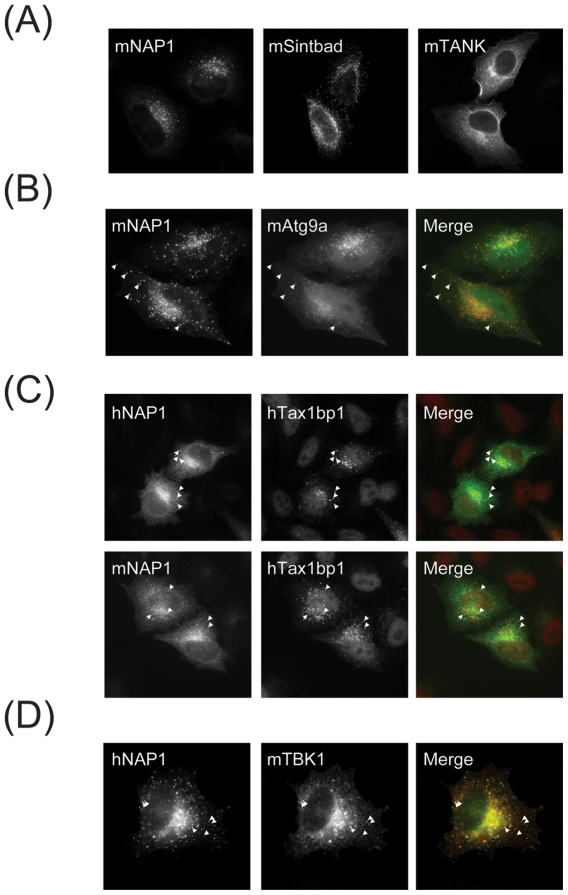
NAP1, SINTBAD and TANK display distinct localization patterns. HeLa cells were transiently transfected with the indicated constructs and stained as described under Materials and Methods. (A) V5-tagged NAP1, SINTBAD and TANK. (B) Co-expression of V5 tagged NAP1 (red) and Atg9a-GFP (green). (C) Co-expression of human or murine V5-tagged NAP1 (green) with myc-tagged Tax1bp1 (red). (D) Co-expression of V5-tagged NAP1 (red) with HA-tagged TBK1 (green).

To further substantiate the hypothesis that the TBK1 adaptors may form alternative complexes, we overexpressed NAP1, Sintbad and TANK in HeLa cells and visualized their subcellular localization by immunofluorescence microscopy (Fig. 2A). While the TANK staining was diffusely perinuclear, NAP1 and Sintbad both displayed a punctate staining which is in agreement with previous reports on NAP1 localization [32]. A recent report has implied peroxisomes as antiviral signaling platforms [33], prompting us to investigate if the NAP1-positive structures could be costained by the peroxisomal marker PTS1 (peroxisomal targeting sequence 1)-DsRed. In addition, we performed a series of cotransfections with established markers for different cell organelles, including lysosomes (Rab7), Endoplasmatic Reticulum Golgi intermediate compartment (ERGIC/p58), the Golgi apparatus (Arf1) or the plasma membrane (Arf6). Our analysis showed that NAP1 was not colocalized with any of those markers (Figure S2).

Upon stimulation with double-stranded DNA, TBK1 has been shown to relocalize to vesicular structures that contain the autophagosome marker Atg9a [34]. We tested whether the NAP1 staining would overlap with the Atg9a staining. Indeed, we found that a subpopulation of NAP1 was colocalized with Atg9a (Fig. 2B). Tax1bp1 which we found associated with TBK1, IKK-i, NAP1 and Sintbad (but not TANK; Fig. 1C) was also found to colocalize with NAP1 (Fig. 2C). TBK1, if coexpressed with NAP1, changed its subcellular localization from diffusely cytosolic to punctuate (data not shown). TBK1 speckles were found to largely colocalize with NAP1 (Fig. 2D). Overall, these data lend support to the hypothesis that the three adaptors reside in different subcellular compartments where they may act as recruiting adaptors for TBK1.

**Figure 3:**
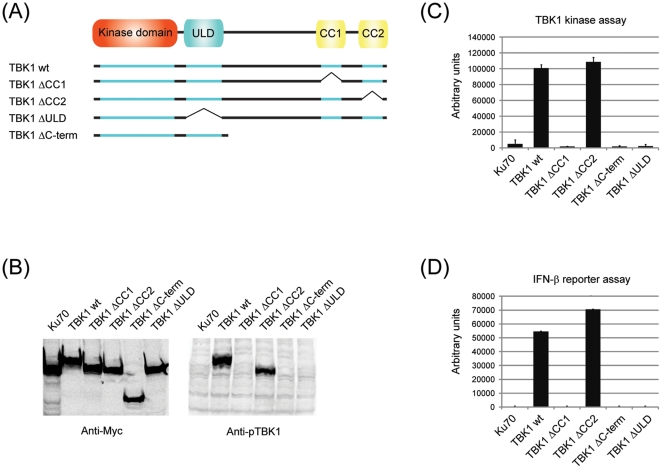
Structural requirements for TBK1 activity. (A) (A) Individual domains of murine TBK1 were either truncated or deleted as indicated in the schematic (kinase domain 1–297, ubiquitin-like domain 304–382, coiled coil 1 619–657, coiled coil 2 682–713). Myc-tagged TBK1 wt or deletion mutants (schematically represented in (A)) were expressed as indicated by transient transfection of 293T cells. Lysates were analyzed by immunoblotting for phospho-TBK1 (B) or submitted to immunoprecipitation using anti-Myc agarose (C). Immunoprecipitates were incubated with a biotinylated IRF3-derived peptide (DLHISNSHPLSLC) in the presence of [γ^32^-P]ATP and incorporated radioactivity was quantified as described in the methods section. (D) Myc-tagged TBK1 wt or deletion mutants were expressed as indicated by transient transfection of 293T cells in the presence of pIFN-Luc and pRL-TK (Promega). Lysates were analyzed using the DualGlo Luciferase Assay System (Promega). Results were normalized to Ku70-induced IFN-β induction.

**Figure 4:**
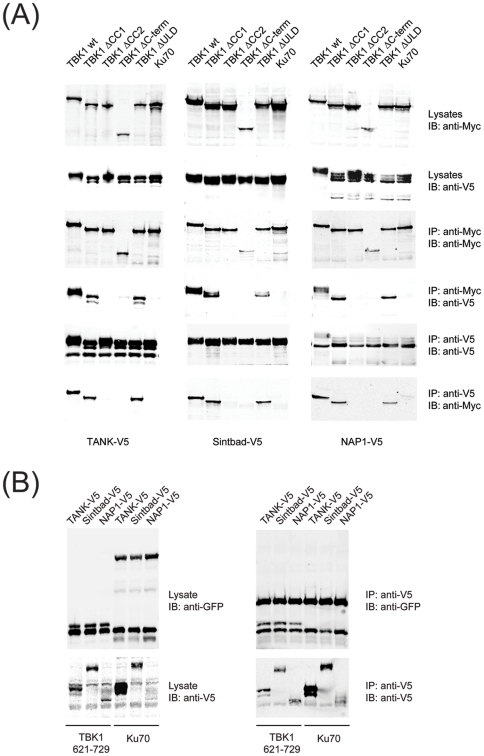
TANK, Sintbad and NAP1 all bind to the coiled-coil 2 region in TBK1. Myc-tagged TBK1 wt or deletion mutants were coexpressed with V5-tagged TBK1 adaptors as indicated by transient transfection of HEK293 cells. Lysates were subjected to immunoprecipitation using anti-Myc agarose (Sigma) or anti-V5 agarose (Sigma). Lysates and eluates were analyzed by immunoblotting for anti-Myc-IRDye800 (Rockland) or anti-V5 (Invitrogen).

TBK1 comprises an N-terminal kinase domain that is flanked by a ubiquitin-like domain (ULD) [35] which has been suggested to form an intramolecular interaction required for catalytic activity. The C-terminal portion of TBK1 contains two coiled-coils whose function is unclear. We created a series of truncation and deletion mutants (Fig. 3A) and assessed the consequences on TBK1 activity at the level of autophosphorylation on Ser 172 (Fig. 3B), kinase activity (Fig. 3C) and transcriptional activation of the IFN-β promoter (Fig. 3D). In all three read-outs, we observed a similar picture: While TBK1 wt and TBK1ΔCC2 were active, all other mutants were completely inactive. The impairment of the ULD deletion mutant has been reported earlier and has been rationalized by an intramolecular interaction between the kinase domain and the ULD that is required for catalytic output [35]. The interaction of TANK, Sintbad and NAP1 with TBK1 was shown to depend on the TBK1-binding domain (TBD) that is shared by all three adaptors [14]. In contrast, it is not known which region in TBK1 is required for adaptor binding. We therefore assessed the requirements for TBK1 adaptor binding using the various deletion and truncation mutants eluded to earlier (Fig. 3A). TANK, Sintbad and NAP1 were specifically associated with TBK1, but not with the negative control Ku70 (Fig. 4A). Binding of TANK, Sintbad and NAP1 was selectively abrogated in mutants lacking the coiled-coil 2 (Fig. 4A), indicating that TANK, Sintbad and NAP1 associate with the same region in TBK1, namely the coiled-coil 2. In addition, fusion of the C-terminus of TBK1 (amino acids 621–729) to GFP was sufficient to artificially induce TBK1 adaptor binding (Fig. 4B), strongly suggesting that the C-terminal coiled-coil 2 is the TBK1 adaptor binding domain. Given the fact that deletion of the coiled coil 2 does not impair TBK1 function upon overexpression (Fig. 3B–D), the TBK1 adaptors seem to be dispensable under these experimental conditions.

**Figure 5:**
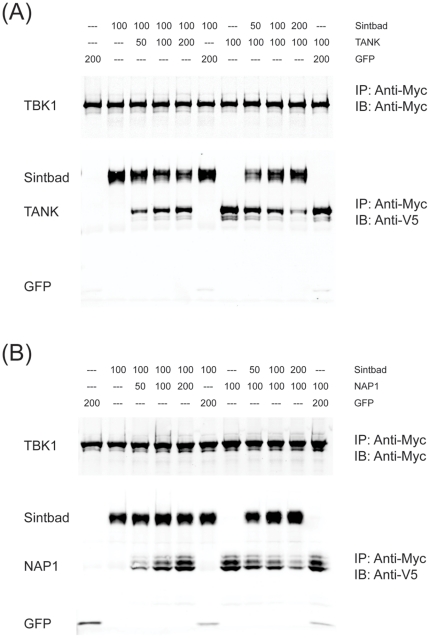
Sintbad competes with TANK or NAP1 for TBK1 binding. (A,B) Individual proteins (Myc-tagged TBK1, V5-tagged GFP, TANK, Sintbad or NAP1) were expressed in HEK293 cells by transient transfection. Cell extracts were mixed as indicated (numbers represent the volume (µl) of each cell extract in each condition) and subjected to immunoprecipitation using anti-Myc agarose (Sigma). Eluates were analyzed by immunoblotting for anti-Myc-IRDye800 (Rockland) or anti-V5 (Invitrogen).

If all TBK1 adaptors associate with the same region in TBK1, this would imply that they either bind simultaneously or compete for binding of the coiled-coil 2 in TBK1. As our previous data (Fig. 1) argued against the former, we tested whether the latter was true by incubating TBK1 with increasing amounts of one adaptor in the presence of constant amounts of the other adaptor. We observed that increasing amounts of Sintbad decreased the amount of TANK associated with TBK1 (Fig. 5A). Similar observations were made for NAP1 (Fig. 5B) which was also competed away by Sintbad. Reduced Sintbad binding was more evident in the presence of increasing amounts of TANK (Fig. 5A) than of NAP1 (Fig. 5B), but NAP1 was also much lower expressed than Sintbad. Overall, these data lend support to a model of mutually exclusive binding of the adaptors to TBK1.

**Figure 6:**
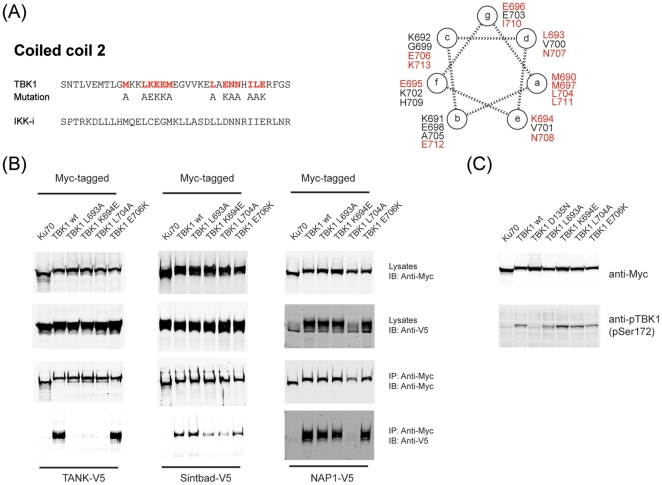
Point mutants in the coiled-coil 2 region of TBK1 lead to selective impairment in adaptor binding. (A) Schematic representation of the coiled-coil 2 region in TBK1 and IKK-i. A helical wheel representation of the coiled coil 2 (M690-E712) was generated according to [36] (B) TBK1 wt or corresponding point mutants in the coiled-coil 2 region were expressed in HEK293 cells, subjected to immunoprecipitation and analyzed as in Fig. 3. (C) TBK1 wt or corresponding point mutants were expressed in HEK293 cells and analyzed for (auto-)phosphorylation at Ser172 by immunoblotting.

In order to study the role of the three TBK1 adaptors in TBK1 signaling, we aimed at creating point mutants that would selectively disrupt binding of each adaptor to TBK1. The position of each amino acid in the coiled-coil 2 of TBK1 is indicated in a model [36] (Fig. 6A). Overall, TANK binding was most susceptible to mutation of the coiled-coil 2 with six mutations (M690A, L693A, K694E, L704A, N708A, L711A) affecting TANK binding (Figure S3A). Sintbad binding was affected by two mutations (K694E, L704A; Figure S3B) while NAP1 was only affected by a single mutation (L704A; Figure S3C). We therefore focused our attention on four mutants: L693A which selectively abrogated TANK binding, K694E which neither bound TANK nor Sintbad, but retained NAP1, L704A which did not bind any TBK1 adaptor and E706K which contained a charge-swap mutation in coiled-coil 2 that did not affect binding of any of the TBK1 adaptors (Fig. 6B). All of the 13 point mutants were able to trigger type-I interferon production upon overexpression (Figure S4), indicating that none of the point mutants were grossly altered or misfolded. In line with this, all four TBK1 point mutants that were selected for further analysis (L693A, K694E, L704A and E706K) were phosphorylated on Ser172 in the activation loop upon overexpression (Fig. 6C), indicating that all of them were catalytically active and able to autophosphorylate once overexpressed.

**Figure 7:**
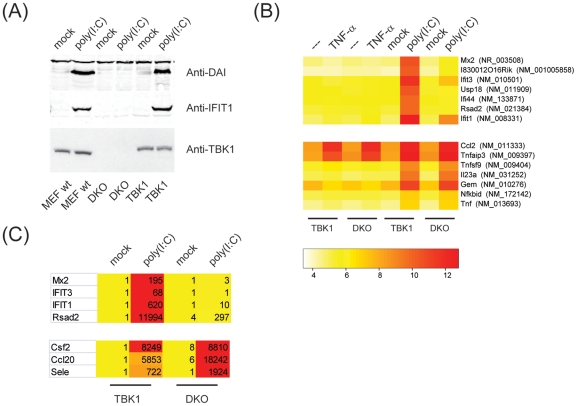
A molecular signature for TBK1. (A) Different batches of MEFs (MEF wt, TBK1/IKK-i-deficient MEFs (DKO for double-KO) reconstituted with TBK1 wt or left untreated) were stimulated with transfected poly(I∶C) for 16 h. Lysates were analyzed by immunoblotting for anti-DAI (Millipore), anti-IFIT1 or anti-TBK1 (Cell Signaling). (B) DKO MEFs reconstituted with TBK1 or left untreated were stimulated with 10 ng/ml TNF-α or 10 µg/ml poly(I∶C) for 4 h. Total RNA was extracted and global changes in transcription were monitored by microarray analysis. Genes were clustered according to recurring patterns. (C) Total RNA obtained for cells stimulated with poly(I∶C) was reverse transcribed and corresponding cDNA levels were analyzed by qPCR. Numbers represent fold changes over TBK1/mock.

TBK1 has been identified as NF-κB-activating kinase (NAK) [37], but the role of TBK1 in NF-κB activation has remained controversial. To properly define TBK1-dependent read-outs, we made use of mouse embryonic fibroblasts (MEFs) that were deficient for TBK1 and IKK-i [3] and reconstituted with TBK1 wt. Reconstitution rescued the production of the interferon-inducible proteins DAI and IFIT1 in response to poly(I∶C), while cells that lacked TBK1 and IKK-i (double-knockouts; DKO) were completely impaired (Fig. 7A). Importantly, TBK1 levels in reconstituted DKO MEFs were comparable to wt MEFs (Fig. 7A), indicating that reconstitution did not result in artificial overexpression.

Next, we aimed at defining a genetic signature for TBK1. To this end, we stimulated DKO MEFs and DKO MEFs reconstituted with TBK1 with TNF-α or poly(I∶C) for 4 h, isolated total RNA and subjected it to microarray analysis. We included TNF-α because embryonic lethality of TBK1-deficient mice can be rescued by crossing those mice into TNFR1 −/− background [4] and because TBK1 has recently been shown to be activated by TNF-α stimulation [38], indicating that TBK1 may either affect TNF-α production or signaling.

When analyzing global changes in gene expression in response to TNF-α or poly(I∶C) stimulation, we found that poly(I∶C)-induced genes can be clustered in a TBK1-dependent and a TBK1-independent group (Fig. 7B). The TBK1-dependent set of genes is induced by poly(I∶C) in cells expressing TBK1, but not in DKO MEFs. It comprises many Interferon-inducible genes such as Mx2, IFIT1 and IFIT3. This set of genes is not induced by TNF-α. In contrast, a second set of genes was induced by poly(I∶C) irrespectively of the presence or absence of TBK1 and was therefore termed TBK1-independent. Many of those TBK1-independent genes are also TNF-α target genes. This suggests that in MEFs, poly(I∶C) stimulates two branches of signal transduction, one of which triggers TNF-α target genes in a TBK1-independent manner while the other one triggers TNF-α-independent genes in a TBK1-dependent manner. We speculate that the former are mostly driven by the NF-κB and AP-1 families of transcription factors, while the latter are likely to be IRF target genes. We confirmed the patterns observed in the microarray analyses by qPCR (Fig. 7C), thereby establishing a TBK1 signature which we used for our further analysis of TBK1 function.

What is the functional consequence of mutation of the coiled-coil 2 region and the associated loss of adaptor binding? Although coiled-coil 2 mutants showed no apparent defect in TBK1 activity upon overexpression, this did not preclude an impact of those mutations on TBK1 function once TBK1 was expressed at physiological levels and activated by upstream stimuli. We therefore used DKO MEFs deficient for TBK1 and IKK-i and reintroduced TBK1 wt, TBK1 D135N (kinase dead) and the coiled-coil mutants eluded to earlier (L693A, K694E, L704A, E706K). We repeatedly failed to generate a cell line that stably expressed the L704A mutant (Figure S5), arguing that this mutant is instable. As the L704A mutant is the only coiled-coil 2 mutant that was defective in NAP1 binding, this may possibly suggest that NAP1 is required for TBK1 stability.

**Figure 8:**
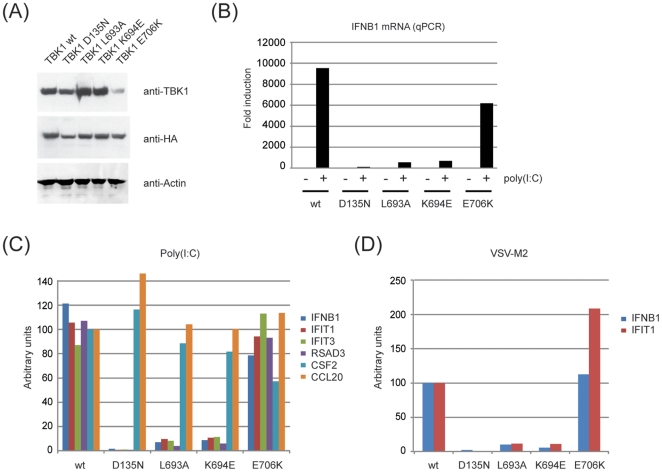
Coiled-coil 2 mutants of TBK1 fail to reconstitute TBK1-/IKK-i-deficient cells. TBK1/IKK-i-deficient MEFs were reconstituted with Strep-HA fusions of TBK1 wt, D135N, L693A, K694E and E706K. (A) Lysates from reconstituted cells were analyzed for immunoblotting using anti-TBK1 (Cell Signaling), anti-HA.11 (Covance) and anti-Actin (Cytoskeleton). (B) Cells as described in (A) were transfected with 10 µg/ml poly(I∶C) for 4 h. Total RNA was extracted, reverse transcribed and analyzed for the IFN-β mRNA by qPCR. Data are represented as fold changes over unstimulated TBK1 wt cells. (C) cDNA from (B) was analyzed by qPCR for the following mRNAs: IFN-β, IFIT1, IFIT3, RSAD3, CSF2, CCL20. Data were normalized such that the average values obtained for TBK1 wt and TBK1 E706K were arbitrarily defined as 100. (D) Cells as described in (A) were infected with Vesicular Stomatitis Virus M2 for 8 h. Total RNA was extracted and processed as described in (B).

When comparing TBK1 levels in reconstituted MEFs, we found that all TBK1 constructs were expressed to similar levels (Fig. 8A; Figure S6). In addition, the binding pattern for endogenous TANK, the only adaptor for which we had an antiserum available, was very similar in the reconstituted MEFs in comparison to the situation of transient overexpression (Figure S5), arguing that the binding patterns observed previously are recapitulated in the reconstituted DKO MEFs.

Stimulation of DKO MEFs reconstituted with TBK1 wt lead to a robust induction of IFN-β mRNA levels that was not seen in unstimulated cells (Fig. 8B), indicating that reconstituted TBK1 is not active in the absence of poly(I∶C) stimulation. TBK1 signaling was dependent on its kinase activity as the kinase-dead TBK1 D135N failed to reconstitute function (Fig. 8B). Mutants lacking the ability to bind TANK (L693A) or TANK/Sintbad (K694E) were strongly impaired in terms of IFN-β induction (Fig. 8B). In contrast, the TBK1 mutant which is unaltered in terms of TBK1 adaptor binding (E706K) showed IFN-β induction comparable to TBK1 wt (Fig. 8B). Similar data were obtained using the previously established TBK1 gene signature: While TBK1 wt and E706K lead to pronounced induction of TBK1 target genes IFIT1, IFIT3 and Rsad3, neither TBK1 D135N, TBK1 L693A nor TBK1 K694E were able to reconstitute TBK1 signaling (Fig. 8C). Nonetheless, TBK1-independent gene expression (Csf2, Ccl20; Fig. 8C) was normal in those cells. Similar observations were made when assessing interferon responses to viral infection using VSV-M2 as a trigger (Fig. 8D). This indicates that mutations in the coiled-coil 2 which affect TBK1 adaptor binding have a profound impact on type-I interferon production.

Altogether, our data suggest that TBK1 does not form a single large multiprotein complex containing TANK, Sintbad and NAP1, but rather forms alternative complexes with each adaptor. We propose that TANK, Sintbad and NAP1 act as adaptors anchoring TBK1 to different subcellular compartments. The interaction of TBK1 to its adaptors is not required for TBK1 activity under overexpression conditions, but is critical for physiological TBK1 activation by upstream stimuli.

## Discussion

In this study, we first aimed at the characterization of the TBK1 protein interaction network to then go beyond its description to a functional dissection. Because of the large scope of the endeavor, we focused here on three main aspects: (i) the structure-function analysis of the central node TBK1 to understand the structural requirements for TBK1 activity, (ii) the relationship of the TBK1 adaptors with TBK1 and with one another and (iii) the functional consequences of disrupting the TBK1 complex architecture. We find that deletion of the ULD and the CC1 region abolishes catalytic output. The TBK1 adaptors, while dispensable for TBK1 activity upon overexpression, were strictly required for TBK1 activation by upstream stimuli.

The protein-protein interaction network around TBK1, IKK-i and their adaptors TANK, Sintbad and NAP1 provides several links to novel and intriguing interactors. Among those, we find AI607873, a murine homologue of the DNA sensor AIM2. A human homologue of AIM2 named IFI16 has recently been proposed to function as a DNA sensor upstream of TBK1 [39]. In the murine genome, the situation is much more complex as the murine HIN200 family comprises 10 members (for a phylogenetic tree see Figure S1A). Among those, we only find AI607873 associated with IKK-i. AI607873 clearly has an affinity for immunostimulatory DNA as it can be precipitated using immobilized double-stranded DNA (Figure S1B). When overexpressed in HeLa cells, AI607873 is predominantly nuclear (Figure S1C), much like many other HIN200 family members [27]. We therefore speculate that either a minor proportion of AI607873 remains cytosolic in macrophages (as has been proposed for human IFI16 [39]) or that AI607873 may be able to translocate to the cytosol under certain physiological conditions. Future work will have to address the function of AI607873 in innate DNA recognition.

When expressing a tagged version of TBK1 in cells, we observed that endogenous TBK1 coprecipitated exogenous tagged TBK1 (Fig. 1A and D). Coprecipitation was almost stoichiometric, suggesting that TBK1 may function as a homodimers or homooligomer. IKK-i was also found associated with TBK1 in our mass spectrometry data (Fig. 1B), but at sub-stoichiometric levels that were too low to be detected by immunoblotting (Fig. 1A). This could indicate that a fraction of TBK1 may form heterodimers with IKK-i.

We speculate that TBK1 serves distinct functions in different signal transduction pathways and that each of those functions is mediated by a different adaptor. Although mouse embryonic fibroblasts are widely used for functional studies in innate immunity, they only express a limited repertoire of innate immune sensors and are thus only responsive to a restricted set of innate immune ligands. We assessed responsiveness of our MEFs to various synthetic ligands and found that poly(I∶C) was the only ligand that robustly triggered type-I interferon responses in those cells (data not shown). Poly(I∶C) responses, however, were completely dependent on TANK binding as both TBK1 coiled-coil 2 mutants that were deficient for TANK binding (L693A and K694E) were severely compromised in terms of interferon induction. As we were unable to derive point mutants with selective defects for Sintbad or NAP1 binding, we could not assess the function of these adaptors in type-I interferon responses. Recent evidence has implicated TBK1 in several other pathways, most importantly the sensing of ubiquitin-coated bacteria [32], [40] and the RalGEF-RalB-Sec5 effector pathway leading to Akt activation [41], [42]. In future, it will be interesting to assess the contribution of the individual adaptors to the non-canonical TBK1 pathways.

We provide several lines of evidence that the TBK1 adaptors do not co-occur in one complex, but rather form alternative complexes. Nevertheless, when analyzing the binding patterns of TANK, Sintbad and NAP1 for the different TBK1 coiled-coil 2 mutants, we observe an interesting hierarchical logic among the mutants: All NAP1-deficient mutants (L704A) are also deficient for Sintbad and TANK binding and all Sintbad-deficient mutants (K694E) are also deficient for TANK binding. TANK is the only adaptor for which point mutants could be isolated that are selectively abrogated in binding of a single adaptor. This may reflect different affinities of TANK, Sintbad and NAP1 for TBK1: While NAP1 is most tightly connected to TBK1 and hence least susceptible to mutation of coiled-coil 2, TANK may have a lower affinity for TBK1 and is therefore highly susceptible to mutation of coiled-coil 2.

Given the fact that TANK-deficient mice have no apparent phenotype with regard to type-I interferon production [17], our finding that the TBK1 mutant L693A in which TANK binding is selectively impaired has a profound defect in IFN-β production is intriguing. We see several possible explanations for this apparent discrepancy: First, the studies on TANK-deficient mice were not done in MEFs, but mostly in cells of hematopoietic origin. Hence, TANK may have a cell-type specific role in MEFs that is not evident in bone marrow-derived macrophages or dendritic cells. Second, TANK may be specifically required for certain stimuli. Although we find that all stimuli that elicit type-I IFN responses in our MEFs are blunted in case of the TBK1 L693A mutant, we cannot formally exclude that there is a stimulus which would be able to trigger type-I IFN responses in a TANK-independent manner. Third, we were using cells deficient for both TBK1 and IKK-i for the reconstitution experiments to avoid the confounding effect of potentially redundant IKK-i. Although we believe that it is unlikely that the requirement for TANK is dependent on the presence or absence of IKK-i, we cannot exclude that the lack of IKK-i sensitizes the cells in such a way that TANK – while dispensable in the presence of IKK-i – suddenly becomes rate-limiting. Fourth, although L693A is a single-amino acid exchange and hence quite a subtle alteration, we cannot formally exclude that other binding partners of TBK1 are also affected by this mutation. Hence, what we observe for the L693A mutant, may be a more complex molecular event than originally anticipated.

TBK1 was originally identified as NF-κB activating kinase (NAK) [37]. We also observe that overexpression of TBK1 strongly triggers NF-κB transcriptional activity in reporter-based assays (data not shown), yet when comparing global changes in transcription between TBK1-expressing and TBK1-deficient cells, we did not find significant differences with regard to NF-κB target genes (Fig. 6B). This indicates that TBK1 has no a major role in promoting NF-κB activation, but is rather restricted to activation of the IRF pathway. Nevertheless, we cannot rule out that the involvement of TBK1 in NF-κB responses is restricted to certain stimuli or cell types we have not studied.

The molecular events underlying TBK1 activation remain poorly understood. Our data provide a first detailed structure-function analysis with the following key conclusions: Deletion of the ubiquitin-like domain [35] or the coiled-coil 1 region in TBK1 lead to a severe impairment in TBK1 function even upon overexpression (Fig. 3). This indicates that deletion of those two domains has profound functional consequences, resulting in a TBK1 molecule that is completely dysfunctional. Deletion or mutation of the coiled-coil 2 has a more subtle phenotype: While coiled-coil 2 mutants are still able to produce an active TBK1 kinase upon overexpression, none of those mutants were active when expressed at endogenous level and activated by exogenous upstream stimuli like poly(I∶C). This indicates that while the coiled-coil 2 is dispensable for overexpression-induced activation, it is strictly required for activation as it occurs under physiological circumstances. The fact that the phenotype of the different coiled-coil 2 mutants could be linked to loss of adaptor binding suggests that the TBK1 adaptors have an important role in the process of TBK1 activation and are not just molecular bystanders.

## Materials and Methods

### Materials

The following resins were used for tandem affinity purification and immunoprecipitation: Rabbit IgG Sepharose (Sigma Aldrich), UltraLink Immobilized Streptavidin Plus Gel (Pierce), Anti-Myc Agarose (Sigma Aldrich), Anti-HA Agarose (Sigma Aldrich). The following commercially available antibodies were used for immunoblotting or immunofluorescence: anti-TBK1 (Cell Signaling), anti-Myc-IRDye 800 (Rockland), anti-Myc (Sigma Aldrich), anti-V5 (mouse monoclonal antibody; Invitrogen), anti-HA (Y-11, Santa Cruz), anti-Tubulin (DM1A; Abcam), anti-DAI (Millipore), anti-Actin (Cytoskeleton). Antibodies against TANK and phospho-TBK1 (phospho-Ser172) were custom-made. Secondary antibodies for immunofluorescence staining (Alexa Fluor 488 and 594 nm) were purchased from Invitrogen.

### Tandem Affinity Purification

Tandem affinity purification was performed as described previously [18]. In brief, RAW264.7 cells were transduced with the respective GS-TAP tagged constructs using retroviral infection. Pools of infected cells were sorted for GFP by FACS, expanded to ∼2×10^9^ cells and subjected to tandem affinity purification. Eluates were subjected to SDS-PAGE and silver staining or immunoblotting.

### Protein Mass Spectrometry

Sample preparation for mass spectrometry (MS) was performed as previously described [18]. Tryptically-digested samples were analysed by liquid chromatography mass spectrometry (LCMS) on a hybrid LTQ Orbitrap XL coupled to an Agilent 1200 series HPLC system as described elsewhere [43]. MS data were searched against the UniProt/SwissProt murine protein database release 2010.09.

### Immunoprecipitation

HEK293 cells were transiently transfected using Polyfect (Qiagen) according to manufacturer's instructions. For expression of TBK1 and TBK1 mutants, we used the plasmid pCS2-N-6Myc which codes for an N-terminal fusion protein of TBK1 with 6× Myc tags. For expression of the TBK1 adaptors, we used the plasmid pTracer C-V5 which codes for a C-terminal fusion protein of the TBK1 adaptors with 1× V5 tag. Cells were harvested 48 h post transfection and lysed in Frackelton buffer (10 mM Tris/HCl pH 7.5, 50 mM NaCl, 30 mM sodium pyrophosphate, 1% Triton X-100, 50 mM NaF and protease inhibitors). Lysates were cleared by centrifugation (13.200 rpm; 10 minutes; 4°C). About 5 mg of lysates (total protein content) were subjected to immunoprecipitation using anti-Myc agarose (Sigma Aldrich) for 2 h at 4°C on a rotary wheel. Precipitates were washed 3× with Frackelton buffer and eluted by boiling in SDS sample buffer.

### Immunofluorescence analysis

For localization studies epitope tagged versions of NAP1, SINTBAD, TANK (all pTracer-C-V5), TAX1BP1 (pCS2-N-6Myc) and TBK1 (pSG-N-4HA-TEV) were used. Atg9a was expressed as a C-terminal fusion protein with GFP. The organelle markers Arf1, Arf6, p58, Rab7 and PTS1 were kind gifts of Jonathan Kagan (Harvard Medical School). HeLa cells were transiently transfected with Fugene6 (Roche) according to manufacturer's instructions. After 24 h, cells were fixed with 2% paraformaldehyde for 20 minutes at room temperature and permeabilized for 10 minutes with 0.1% Triton X-100. Samples were treated with block buffer (2% goat serum and 50 mM ammonium chloride in PBS) for 30 minutes, and the appropriate antibodies were diluted in block buffer. Antibody binding was detected using antibodies conjugated with Alexa Fluor 488 or 594. Samples were imaged with a microscope (Eclipse 80i; Nikon) fitted with a Plan-Fluor 40× objective (Nikon) with a numerical aperture of 0.75. Images were processed with Photoshop (Adobe).

### Kinase assay

For peptide based assays, Myc-TBK1 wt or mutants were obtained from HEK293 cells by immunoprecipitation using anti-Myc agarose (Sigma). Precipitated TBK1 was incubated with a biotinylated peptide derived from IRF3 (DLHISNSHPLSLC) in the presence of 50 µM ATP, 5 µCi [γ-32P]ATP and kinase buffer (40 mM Tris/HCl pH 7.5, 10 mM MgCl_2_, 1 mM DTT) for 30 minutes at 30°C in a 20 µl volume. The reaction was terminated by adding 12.5 µL guanidinium chloride (7,5 M) and the terminated reaction was spotted onto a SAM2 Biotin Capture membrane (Promega, Madison, WI) and further treated according to manufacturer's instructions. Kinase activity was then determined in a gamma spectrometer.

### Retroviral infection

RAW264.7 cells were infected using a pCL-derived retroviral vector as described previously [18]. Mouse embryonic fibroblasts (MEFs) were infected using a pMSCV-based retroviral vector as described previously [44]. In brief, 293 gp cells were seeded on day 1 and transfected with the retroviral vector, together with a VSV-G encoding plasmid. Viruses were harvested by 48 h post transfection, cleared by centrifugation and filtration and applied to MEFs for 24 h. In some cases, the infection was repeated after 24 h to increase the infection efficiency. MEFs were sorted for GFP by FACS to enrich for transduced cells.

### RNA extraction, microarray analysis and qPCR

RNA was extracted from MEFs using the SV Total RNA Isolation System (Promega) according to the manufacturer's instructions. 200 ng of total RNA were then used for microarray analysis. Preparation of terminally-labelled cDNA, hybridization to GeneChip Mouse Gene 1.0 ST Arrays (Affymetrix, Santa Clara, CA) and scanning of the arrays were carried out according to manufacturer's instructions (www.affymetrix.com). Signal extraction and normalization were performed using the RMA algorithm and [45]. Signal intensity reflecting mRNA expression levels was illustrated with heatmaps on log scale according to a color-coded intensity scale using R software (www.r-project.org).

For qPCR, total RNA was reverse transcribed using M-MuLV Reverse Transcriptase (Fermentas) according to the manufacturer's instructions. PCR primers used for quantitative RT-PCR of murine genes include:

IFNB1 (NM_010510) fwd, TCAGAATGAGTGGTGGTTGC

IFNB1 (NM_010510) bwd, GACCTTTCAAATGCAGTAGATTCA

IFIT1 (NM_008331) fwd, CATGGGAGAGAATGCTGATG

IFIT1 (NM_008331) bwd, GTCAAGGAACTGGACCTGCT

IFIT3 (NM_010501) fwd, CCAGCAGCACAGAAACAGAT

IFIT3 (NM_010501) bwd, GAAGGATCGCTTCCAGAGATT

Mx2 (NM_013606) fwd, AAACACAGGCGTTGATTCAG

Mx2 (NM_013606) bwd, TTGTTCACAGACTCCTGGTCTT

Rsad2 (NM_021384) fwd, CAAGCGAGGACTGCTTCTG

Rsad2 (NM_021384) bwd, TGTCCTGAAGGAAGGGTTCT

Csf2 (NM_009969) fwd, GCCTGTCACGTTGAATGAAG

Csf2 (NM_009969) bwd, TTGAGTTTGGTGAAATTGCC

Ccl20 (NM_016960) fwd, GGTACTGCTGGCTCACCTCT

Ccl20 (NM_016960) bwd, AATGTCACAAGCTTCATCGG

Sele (NM_011345) fwd, TTCGTGTACCAATGCATCCT

Sele (NM_011345) bwd, CAAGTCACAGCTTGCTCACA

## Supporting Information

Figure S1 **AI607873 is a murine AIM2 homologue.** (A) Phylogenetic tree of human and murine AIM2 homologs. (B) Myc-tagged AIM2 or TBK1 or V5-tagged AI607873 were overexpressed in HEK293 by transient transfection. Cell extracts were incubated with biotinylated Interferon-stimulatory DNA (ISD), immunobilized on UltraLink Immobilized Streptavidin Plus Gel (Pierce). Bound proteins were washed 3×, eluted in SDS sample buffer and visualized by immunoblotting using anti-Myc-IRDye800 (Rockland). (C) Hela cells were transiently transfected with V5-tagged AI607873 or Myc-tagged TBK1 and stained as described under Materials and Methods.
(TIF)

Figure S2 **NAP1 does not co-localize with membrane-bound organelles.** Immunostaining of HeLa cells transiently co-transfected with V5-tagged NAP1 and a selection of organelle markers, i. e. PTS1-DsRed (peroxisomes, A), Arf1-GFP (Golgi apparatus, B), Arf6-GFP (plasma membrane, C), p58-GFP (ERGIC, D), and Rab7-GFP (lysosomes, E).
(TIF)

Figure S3 **Analysis of TBK1 point mutants in the coiled coil 2 region.** Myc-tagged TBK1 wt and mutants were coexpressed with V5-tagged TBK1 adaptors (TANK in (A), Sintbad in (B) and NAP1 in (C)) as indicated by transient transfection of HEK293 cells. Cell extracts were subjected to immunoprecipitation using anti-Myc agarose (Sigma). Lysates and eluates were analyzed by immunoblotting for anti-Myc-IRDye800 (Rockland) or anti-V5 (Invitrogen).
(TIF)

Figure S4 **IFN reporter assay of TBK1 point mutants.** HEK293 cells were transiently transfected with TBK1 wt or TBK1 point mutants (as indicated), together with pIFN-beta-Luc (Firefly luciferase) and pRL-TK (Renilla luciferase). Cells were harvested 24 h post transfection, lysed in Passive Lysis Buffer and analyzed using the Dual-Glo Luciferase Assay System (Promega). Firefly luciferase levels were normalized to Renilla Luciferase Levels.
(TIF)

Figure S5 **Binding pattern of TBK1 mutants expressed in MEFs.** TBK1/IKK-i-deficient mouse-embryonic fibroblasts (MEFs) were reconstituted with Strep-HA fusions of TBK1 wt or the corresponding mutants by retroviral transduction.Corresponding lysates were subjected to immunoprecipitation using anti-HA agarose (Sigma). Lysates and eluates were analyzed by immunoblotting for anti-HA.11 (Covance), anti-TBK1 (Cell Signaling) or anti-TANK (custom made).
(TIF)

Figure S6 **Epitope mapping for anti-TBK1 antibody.** HEK293 cells were transiently transfected with TBK1 wt or TBK1 point mutants (as indicated). Lysates were analyzed by immunoblotting using anti-Myc-IRDye800 (Rockland) or anti-TBK1 (Cell Signaling).
(TIF)

Table S1 **Mass Spectrometry dataset.** A list of proteins identified by MS in TBK1-related pulldowns. Proteins are divided into 3 groups: baits (name in bold, violet background), specific binders (bold), non-specific binders (regular font). Columns are: UniProt protein accession code (primaryac), name of protein-coding gene as reported by UniProt (genename), UniProt protein name (description), length of protein AA-sequence (seqlength), whether protein was considered specific bait binder or not after filtering (specific), number of different peptides/number of spectras/sequence coverage of protein in pulldown P10XX (pc/sc/seqcov.P10XX), number of unique peptides of a protein (i.e. not shared with any other known protein) in pulldown P10XX (pc_sup.P10XX). The name of the bait corresponding to pulldown P10XX is given in the second row of the table.
(XLS)
