# Supplementary figures and images for: Functional Dissection of the TBK1 Molecular Network
Source: PLoS One. 2011 Sep 8;6(9):e23971. doi: 10.1371/journal.pone.0023971 (PMC3169550; doi:10.1371/journal.pone.0023971)

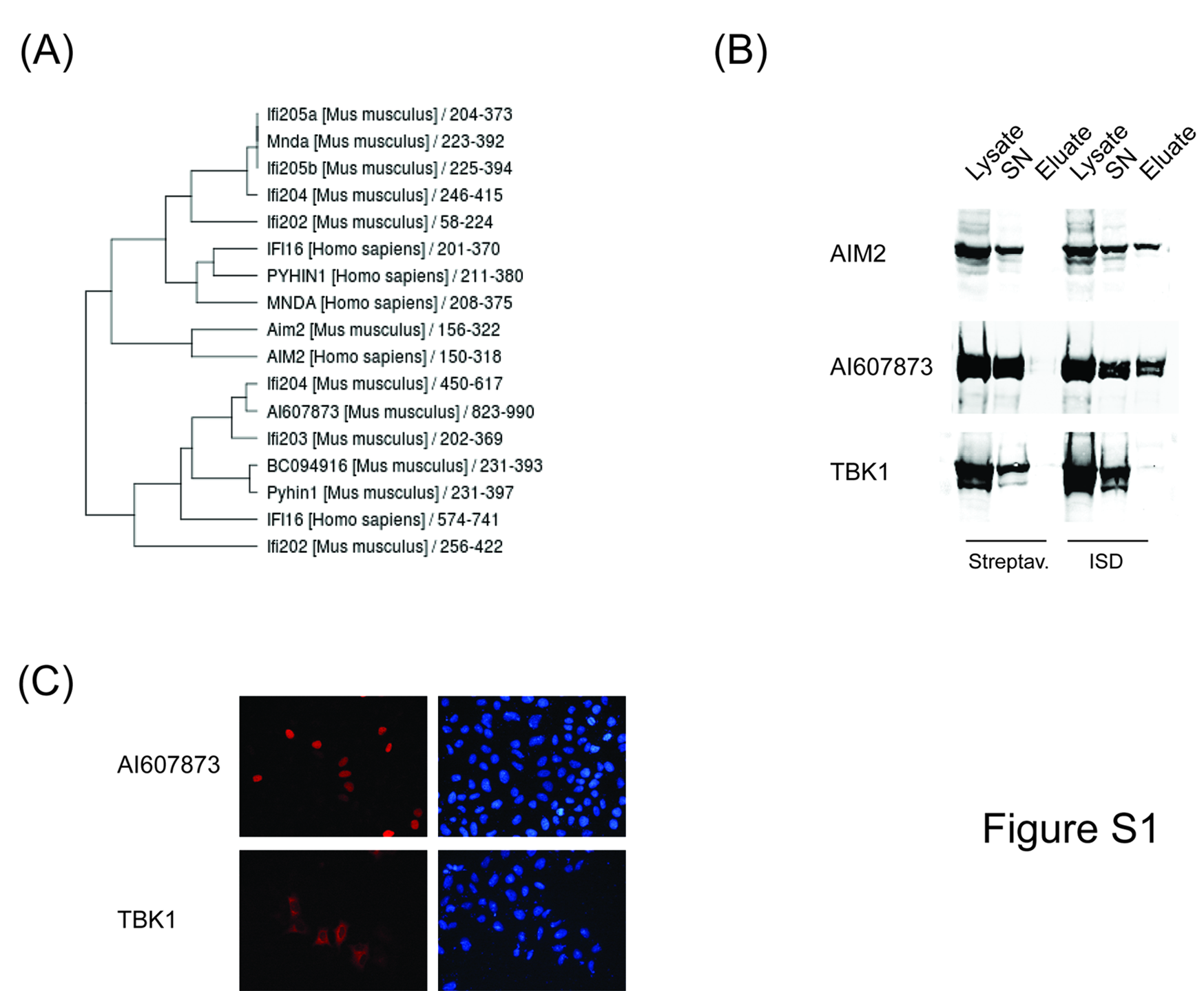

Supplement: Figure S1 — AI607873 is a murine AIM2 homologue. (A) Phylogenetic tree of human and murine AIM2 homologs. (B) Myc-tagged AIM2 or TBK1 or V5-tagged AI607873 were overexpressed in HEK293 by transient transfection. Cell extracts were incubated with biotinylated Interferon-stimulatory DNA (ISD), immunobilized on UltraLink Immobilized Streptavidin Plus Gel (Pierce). Bound proteins were washed 3×, eluted in SDS sample buffer and visualized by immunoblotting using anti-Myc-IRDye800 (Rockland). (C) Hela cells were transiently transfected with V5-tagged AI607873 or Myc-tagged TBK1 and stained as described under Materials and Methods. (TIF) [file pone.0023971.s001.tif]

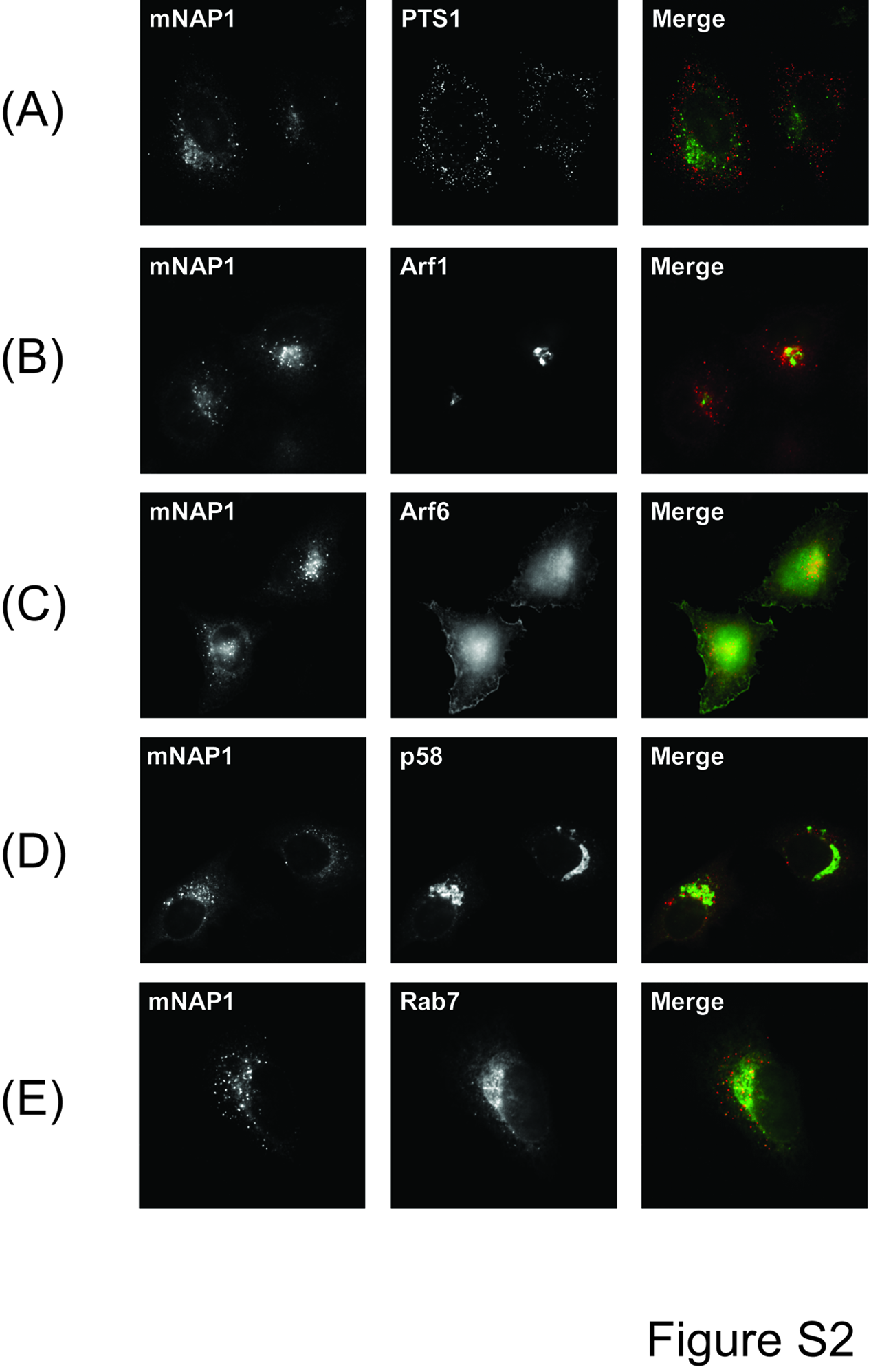

Supplement: Figure S2 — NAP1 does not co-localize with membrane-bound organelles. Immunostaining of HeLa cells transiently co-transfected with V5-tagged NAP1 and a selection of organelle markers, i. e. PTS1-DsRed (peroxisomes, A), Arf1-GFP (Golgi apparatus, B), Arf6-GFP (plasma membrane, C), p58-GFP (ERGIC, D), and Rab7-GFP (lysosomes, E). (TIF) [file pone.0023971.s002.tif]

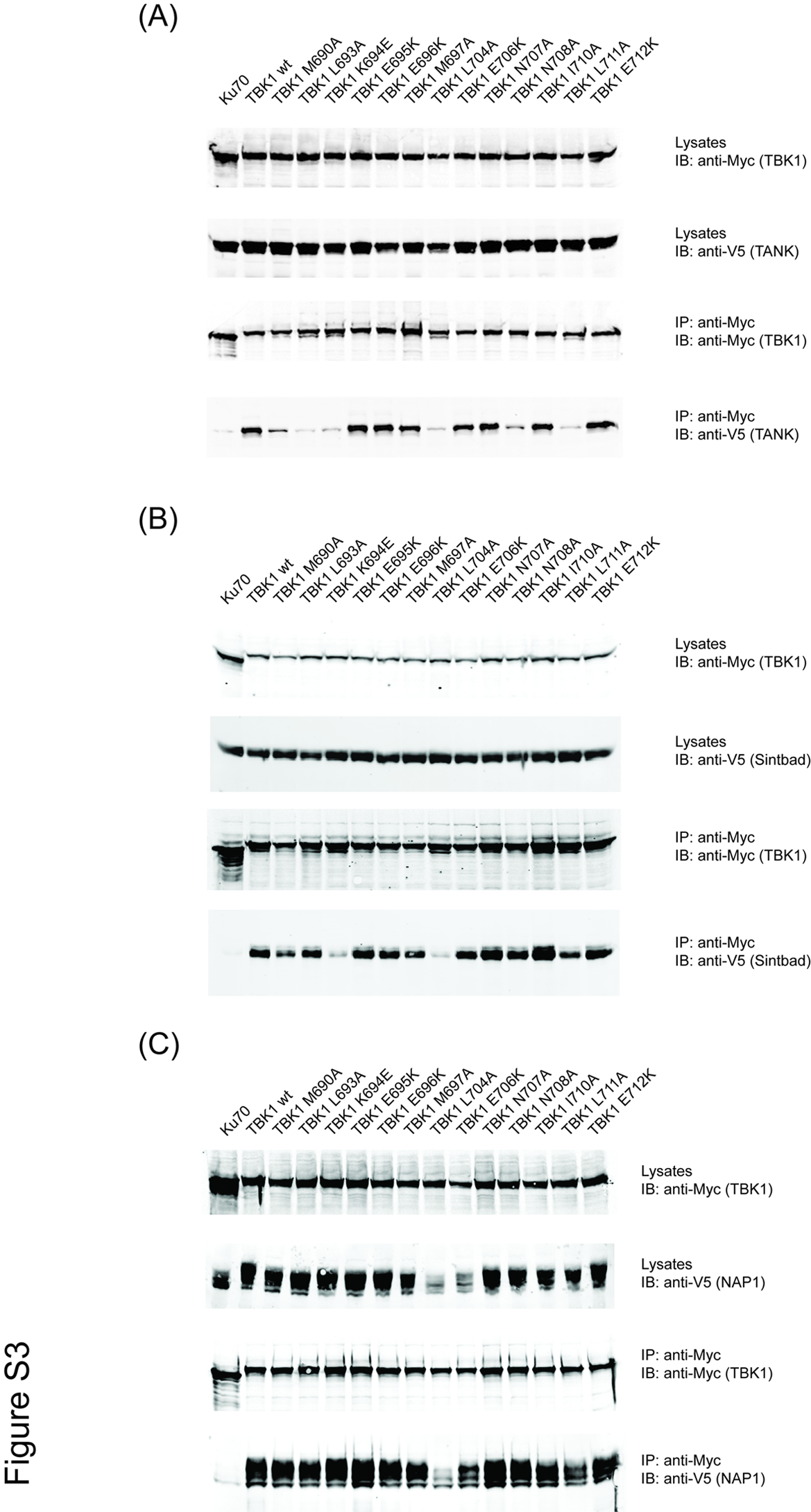

Supplement: Figure S3 — Analysis of TBK1 point mutants in the coiled coil 2 region. Myc-tagged TBK1 wt and mutants were coexpressed with V5-tagged TBK1 adaptors (TANK in (A), Sintbad in (B) and NAP1 in (C)) as indicated by transient transfection of HEK293 cells. Cell extracts were subjected to immunoprecipitation using anti-Myc agarose (Sigma). Lysates and eluates were analyzed by immunoblotting for anti-Myc-IRDye800 (Rockland) or anti-V5 (Invitrogen). (TIF) [file pone.0023971.s003.tif]

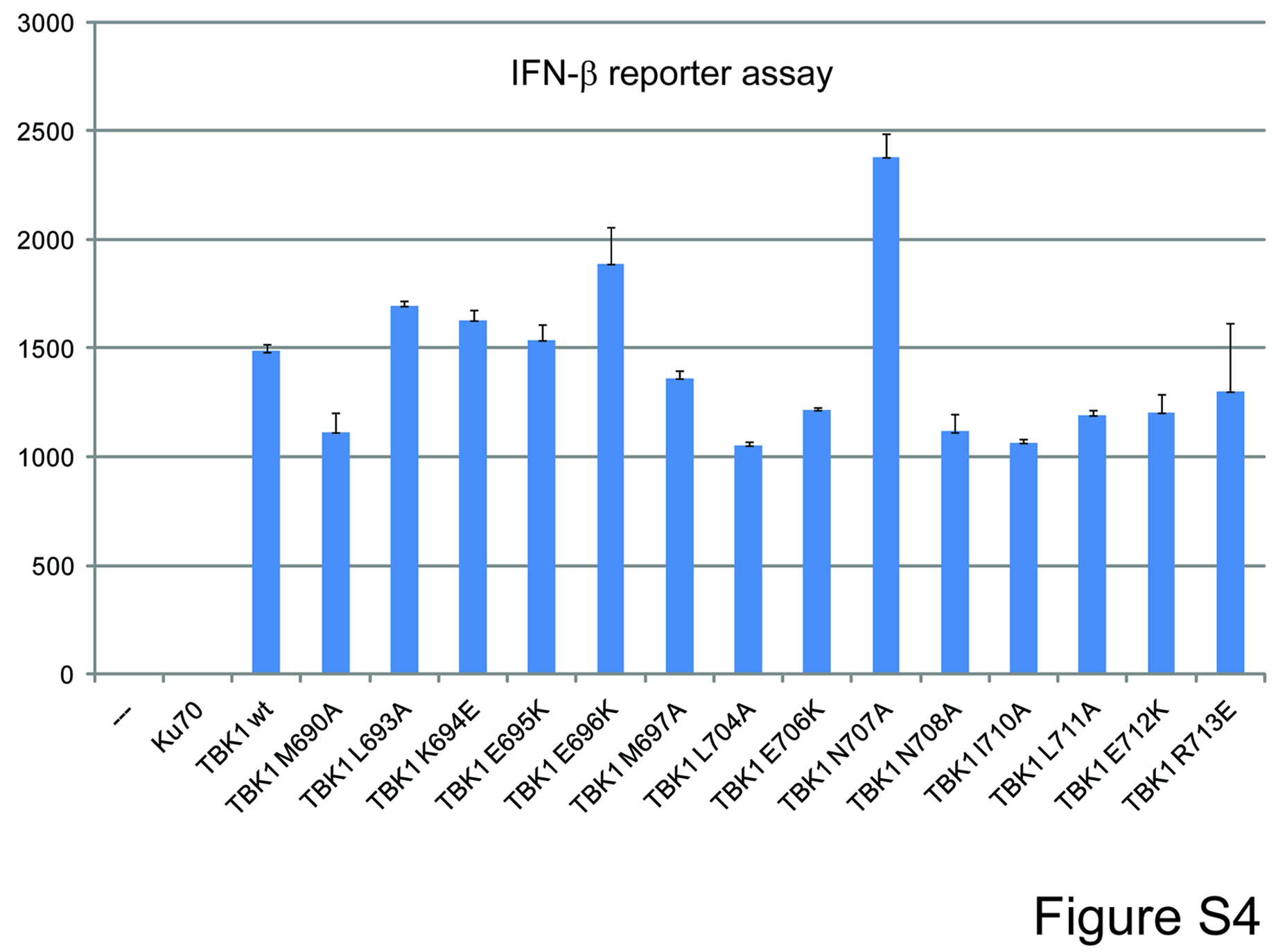

Supplement: Figure S4 — IFN reporter assay of TBK1 point mutants. HEK293 cells were transiently transfected with TBK1 wt or TBK1 point mutants (as indicated), together with pIFN-beta-Luc (Firefly luciferase) and pRL-TK (Renilla luciferase). Cells were harvested 24 h post transfection, lysed in Passive Lysis Buffer and analyzed using the Dual-Glo Luciferase Assay System (Promega). Firefly luciferase levels were normalized to Renilla Luciferase Levels. (TIF) [file pone.0023971.s004.tif]

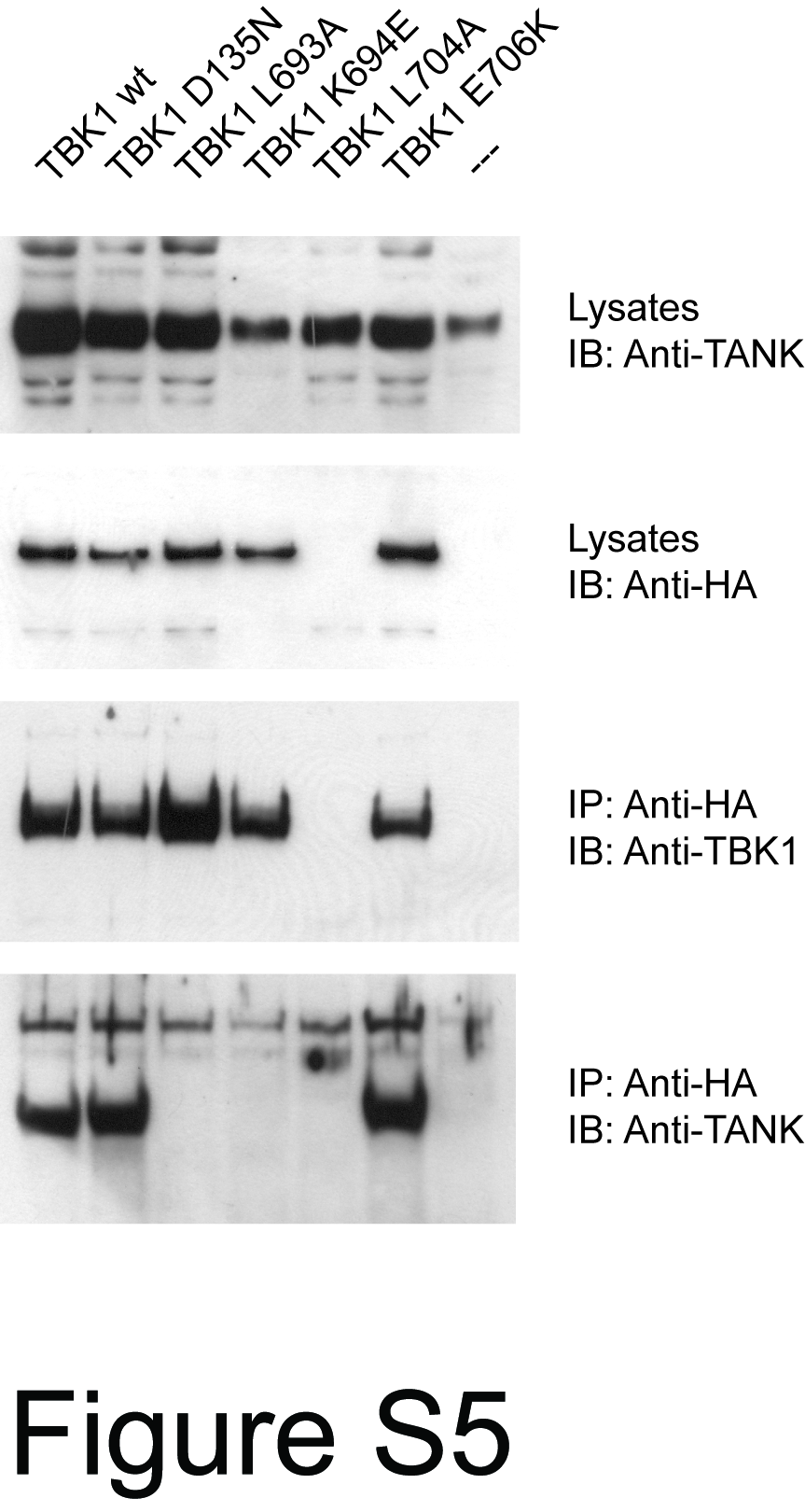

Supplement: Figure S5 — Binding pattern of TBK1 mutants expressed in MEFs. TBK1/IKK-i-deficient mouse-embryonic fibroblasts (MEFs) were reconstituted with Strep-HA fusions of TBK1 wt or the corresponding mutants by retroviral transduction.Corresponding lysates were subjected to immunoprecipitation using anti-HA agarose (Sigma). Lysates and eluates were analyzed by immunoblotting for anti-HA.11 (Covance), anti-TBK1 (Cell Signaling) or anti-TANK (custom made). (TIF) [file pone.0023971.s005.tif]

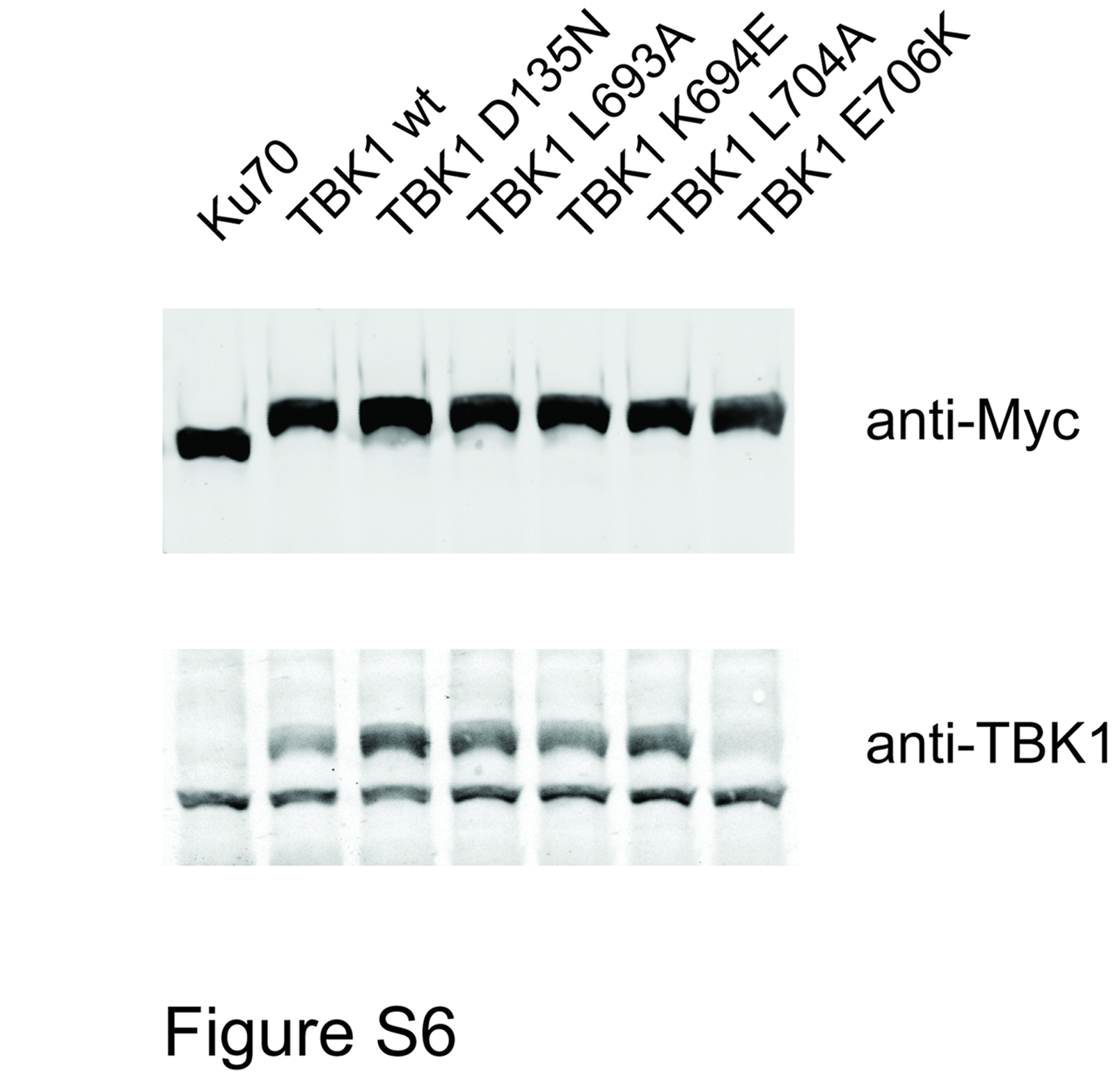

Supplement: Figure S6 — Epitope mapping for anti-TBK1 antibody. HEK293 cells were transiently transfected with TBK1 wt or TBK1 point mutants (as indicated). Lysates were analyzed by immunoblotting using anti-Myc-IRDye800 (Rockland) or anti-TBK1 (Cell Signaling). (TIF) [file pone.0023971.s006.tif]
